# Improving criteria for dissemination in space in multiple sclerosis by including additional regions

**DOI:** 10.1002/acn3.52170

**Published:** 2024-07-30

**Authors:** Michael A. Foster, Giuseppe Pontillo, Indran Davagnanam, Sara Collorone, Ferran Prados, Baris Kanber, Marios C. Yiannakas, Lola Ogunbowale, Ailbhe Burke, Claudia A. M. Gandini Wheeler‐Kingshott, Olga Ciccarelli, Wallace Brownlee, Frederik Barkhof, Ahmed T. Toosy

**Affiliations:** ^1^ Queen Square MS Centre, Department of Neuroinflammation, UCL Queen Square Institute of Neurology, Faculty of Brain Sciences University College London London UK; ^2^ Department of Radiology and Nuclear Medicine Amsterdam UMC, Vrije Universiteit Amsterdam Amsterdam the Netherlands; ^3^ Department of Advanced Biomedical Sciences and Electrical Engineering and Information Technology University of Naples Federico II Naples Italy; ^4^ Department of Brain Repair & Rehabilitation, UCL Queen Square Institute of Neurology, Faculty of Brain Sciences University College London London UK; ^5^ Centre for Medical Image Computing, Department of Medical Physics and Biomedical Engineering, Faculty of Engineering Science University College London London UK; ^6^ Universitat Oberta de Catalunya Barcelona Spain; ^7^ Strabismus and Neuro‐Ophthalmology Service Moorfields Eye Hospital NHS Foundation Trust London UK; ^8^ Department of Brain and Behavioural Sciences University of Pavia Pavia Italy; ^9^ NIHR University College London Hospitals Biomedical Research Centre London UK

## Abstract

**Objective:**

We investigated the effects of adding regions to current dissemination in space (DIS) criteria for multiple sclerosis (MS).

**Methods:**

Participants underwent brain, optic nerve, and spinal cord MRI. Baseline DIS was assessed by 2017 McDonald criteria and versions including optic nerve, temporal lobe, or corpus callosum as a fifth region (requiring 2/5), a version with all regions (requiring 3/7) and optic nerve variations requiring 3/5 and 4/5 regions. Performance was evaluated against MS diagnosis (2017 McDonald criteria) during follow‐up.

**Results:**

Eighty‐four participants were recruited (53F, 32.8 ± 7.1 years). 2017 McDonald DIS criteria were 87% sensitive (95% CI: 76–94), 73% specific (50–89), and 83% accurate (74–91) in identifying MS. Modified criteria with optic nerve improved sensitivity to 98% (91–100), with specificity 33% (13–59) and accuracy 84% (74–91). Criteria including temporal lobe showed sensitivity 94% (84–98), specificity 50% (28–72), and accuracy 82% (72–90); criteria including corpus callosum showed sensitivity 90% (80–96), specificity 68% (45–86), and accuracy 85% (75–91). Criteria adding all three regions (3/7 required) had sensitivity 95% (87–99), specificity 55% (32–76), and accuracy 85% (75–91). When requiring 3/5 regions (optic nerve as the fifth), sensitivity was 82% (70–91), specificity 77% (55–92), and accuracy 81% (71–89); with 4/5 regions, sensitivity was 56% (43–69), specificity 95% (77–100), and accuracy 67% (56–77).

**Interpretation:**

Optic nerve inclusion increased sensitivity while lowering specificity. Increasing required regions in optic nerve criteria increased specificity and decreased sensitivity. Results suggest considering the optic nerve for DIS. An option of 3/5 or 4/5 regions preserved specificity, and criteria adding all three regions had highest accuracy.

## Introduction

Diagnosing relapsing–remitting multiple sclerosis (MS) requires demyelination disseminated in space and time.^1^ Currently, dissemination in space (DIS) is demonstrated on MRI by identifying at least one T2‐hyperintense lesion in at least two of four CNS areas: periventricular, cortical/juxtacortical, and infratentorial brain regions, and spinal cord.^2^ Radiological dissemination in time (DIT) can be achieved through the simultaneous presence of gadolinium‐enhancing and nonenhancing lesions on one scan, or by presence of new lesions on follow‐up scans.^2^

Lesions in other regions are characteristic but not in the current DIS framework, including the optic nerve, corpus callosum, and temporal lobe.^3^ Optic neuritis is a common presenting syndrome^4^ with well‐described imaging appearances.^5^ Demyelinating lesions are seen in optic nerves without a prior history of optic neuritis.^6^, ^7^, ^8^ 2016 MAGNIMS MRI criteria for MS diagnosis recommended including optic nerve as a fifth location for DIS^9^; however, the 2017 McDonald criteria did not include this proposal, citing lack of sensitivity and specificity data for techniques identifying optic nerve lesions.^2^ Several studies have now addressed this^10^: Brownlee et al.^11^ demonstrated increased diagnostic performance when optic nerve involvement (assessed clinically and/or with visual evoked potentials) was considered for DIS; Vidal‐Jordana et al.^12^ described similar findings with visual evoked potentials in a larger cohort; and Bsteh et al.^13^ reported improvement in performance with use of optical coherence tomography. The inclusion of the optic nerve in DIS was also supported by an analysis in a Hispanic population, where optic neuritis is more common.^14^ A recent multicenter analysis has examined the impact of including optic nerve lesions as identified on optic nerve MRI on DIS criteria – it noted an increase in sensitivity with a commensurate loss of specificity as accuracy remained stable.^15^

The corpus callosum is another typical location for demyelinating lesions in MS which can be detected using MRI.^16^ The morphology and topography of callosal lesions can also help distinguish between MS and other demyelinating conditions such as neuromyelitis optica spectrum disorders,^17^, ^18^ or imaging mimics like Susac's syndrome.^19^ Callosal lesions can currently be used to satisfy DIS, but only if abutting a ventricle and classified as a periventricular lesion. Studies investigating the contribution of callosal lesions to predict MS have reported mixed results: Arrambide et al.^20^ and Barkhof et al.^21^ confirmed their presence in MS, but not a contribution to its diagnosis, whereas Jafari et al.^22^ noted that combining the presence of callosal lesions with the Barkhof criteria for MRI assessment^21^ improved prediction of MS diagnosis.

Temporal lobe lesions are observed in MS, especially in the cortex or near the temporal horns of the lateral ventricles.^3^, ^23^ These can only currently be considered for DIS where they are already periventricular or cortical/juxtacortical. Their presence with callosal lesions is predictive of demyelinating activity^24^ and can distinguish MS from neuromyelitis optica spectrum disorders.^25^, ^26^, ^27^ Temporal lobe lesions make a significant contribution to Extended Disability Status Scale outcomes^28^; moreover, people with MS with co‐existing epilepsy^29^ or depression^30^ have a higher temporal lobe lesion load.

Against this background, we present a novel analysis of variations of DIS diagnostic criteria in a single‐center, highly phenotyped cohort with consistent follow‐up to assess their ability to facilitate earlier MS diagnosis after a first demyelinating event. We demonstrate how MRI can establish optic nerve involvement and earlier MS diagnosis.

## Methods

### Participants

The study recruited people presenting with neurological symptoms suggestive of a first episode of demyelination within 3 months of their presentation. Suitable participants were identified at the National Hospital for Neurology & Neurosurgery and at Moorfields Eye Hospital, both in London, UK. All participants had to be aged 18–65 years old, able to give written informed consent in English, and able to have an MRI scan. Participants needed to have clinically isolated syndrome (CIS) without prior diagnosis of MS and no other medical condition that might affect the central nervous system. The local research ethics committee approved the study protocol (13/LO/1762; 13/0231‐CIS2013), and all participants gave written informed consent.

### Clinical and MRI assessment

Participants underwent assessment at baseline and were then followed up at regular intervals (6, 12, 36, and 60 months). New clinical relapse activity was recorded. Participants were assessed for disability using the Expanded Disability Status Scale.^31^ Though not part of the study protocol, some participants underwent CSF examination as part of their routine clinical care. The 2017 revisions of the McDonald criteria^2^ were applied to all participants in the study to establish if MS could be diagnosed at their presentation and at follow‐up visits.

MRI scans were acquired on a 3‐Tesla Philips Achieva TX (Philips, Best, the Netherlands) that during the study was upgraded to a 3T Philips Ingenia CX scanner. All participants underwent imaging of the brain, optic nerve, and spinal cord, including gadolinium‐based contrast agent. Details of MRI protocols are provided in the supplementary materials.

### Image analysis

**Figure 1:**
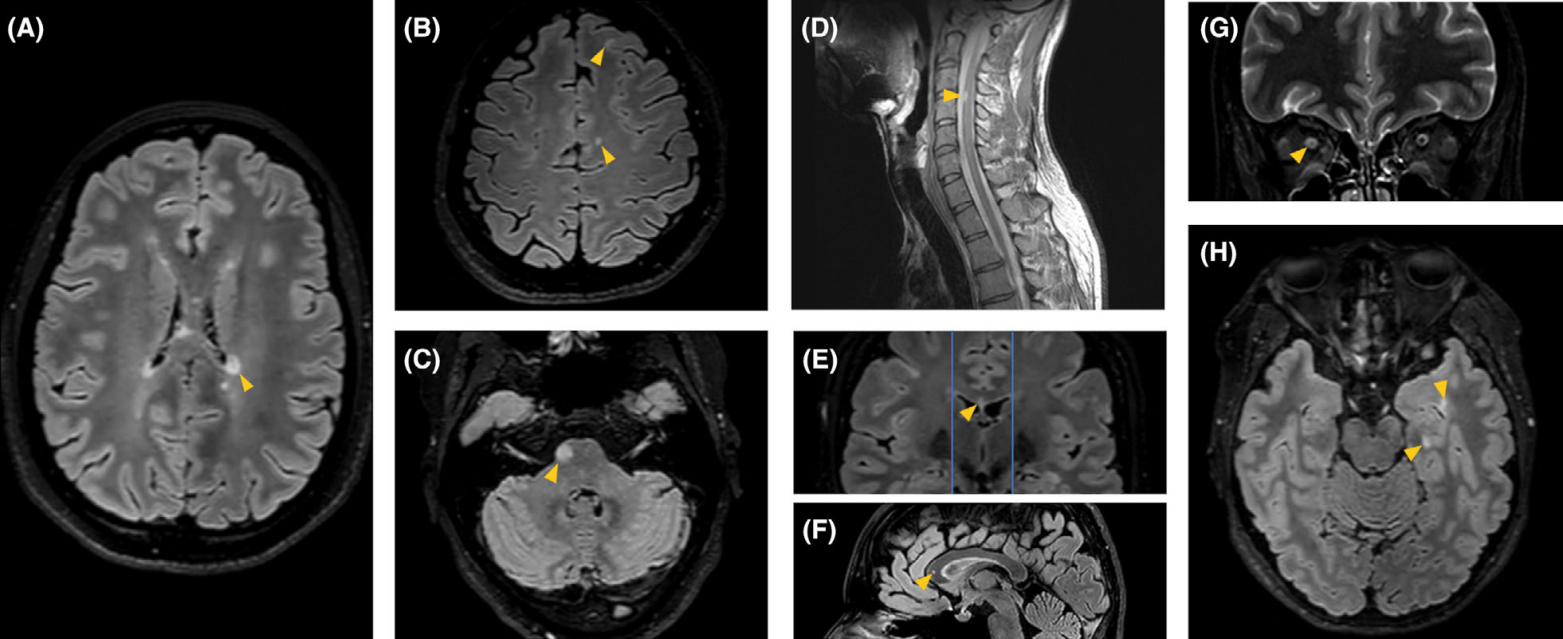
Examples of lesions (arrowheads) in the different compartments: (A) periventricular; (B) juxtacortical; (C) infratentorial; (D) spinal cord; (E, F) corpus callosum; (G) optic nerve; (H) temporal lobe.

Three experienced neuroradiologists (GP, ID and FB) reviewed all images by consensus. Lesions suggestive of demyelination were assessed on baseline scans following previous published guidance for current DIS locations (Fig. 1).^1^ Corpus callosum lesions had to be included within two planes traversing the external walls of the lateral ventricles, regardless of whether they touched the ventricular roof. Temporal lobe lesions could be either cortical/juxtacortical or located in the deep or periventricular white matter. Optic nerve and brain lesions were primarily assessed on coronal short tau inversion recovery and 3D fluid‐attenuated inversion recovery images, respectively, while spinal cord lesions were assessed on sagittal proton density‐weighted and T2‐weighted scans. For all identified lesions, distinct brain regions were considered as mutually exclusive locations – for example, for modified criteria including the corpus callosum as a separate compartment, a lesion in the corpus callosum did not contribute to the periventricular lesion count, or, for modified criteria including the temporal lobe as a separate compartment, a lesion in the juxtacortical temporal white matter did not contribute to the cortical/juxtacortical lesion count.

DIS was defined according to the 2017 McDonald DIS criteria (requiring ≥2/4 regions),^2^ and the following modified criteria including a fifth anatomical location (≥2/5 regions): (i) modified DIS including the optic nerve, (ii) modified DIS including the temporal lobe, and (iii) modified DIS including the corpus callosum. Performance of the modified criteria including the optic nerve was also assessed where 3/5 or 4/5 regions were required to satisfy DIS. Finally, the three new regions (optic nerve, temporal lobe, and corpus callosum) were added to the standard four compartments, with ≥3/7 regions required to demonstrate DIS. Additionally, in patients where postcontrast MRI sequences and/or the oligoclonal band information were available, the combination of modified DIS criteria with DIT was also considered.

### Statistical analysis

The performance of the modified DIS criteria at baseline was tested against the 2017 McDonald criteria (DIS and DIT) for the diagnosis of relapsing–remitting multiple sclerosis at any time during the study – where DIS was satisfied, diagnosis of relapsing–remitting MS was determined by a clinical relapse, new T2 lesion, the simultaneous presence of gadolinium‐enhancing and nonenhancing lesions on MRI, or the presence of CSF oligoclonal bands.^2^ We calculated sensitivity, specificity, accuracy, and positive and negative predictive values (with 95% confidence intervals, CI). The McNemar test was used to quantitatively compare the performance of the modified criteria with that of the 2017 DIS criteria, with a significance level set at *P* < 0.05. Finally, a receiver operating characteristic (ROC) curve analysis was performed to assess the diagnostic accuracy of demonstrating DIS with MRI across the spectrum of possibly involved compartments. Statistical analyses were done using SPSS v25.

## Results

We recruited 84 participants with clinically isolated syndrome between 2014 and 2021 (53 females, age 32.8 ± 7.1 years). Of these, 68 (81%) presented with optic neuritis, 7 with a brainstem/cerebellar syndrome, 5 with a spinal cord syndrome, 3 hemispheric presentations and 1 multifocal presentation. Sixty‐five were recruited from Moorfields Eye Hospital, with the remainder from the National Hospital for Neurology and Neurosurgery. Median time of symptom onset to first MRI was 54 days (interquartile range 42 days). At baseline MRI, 73 participants (87%) had optic nerve lesions, 64 (76%) had temporal lobe lesions, and 52 (62%) had callosal lesions. Of participants with temporal lobe lesions, 61 had lesions in temporal deep white matter; 31 of this group did not have a co‐existing periventricular or cortical/juxtacortical temporal lobe lesion. In participants with callosal lesions, six had lesions not abutting the ventricles; only one of this group did not also have a periventricular callosal lesion. Contrast‐enhanced sequences were obtained in 78 participants (93%); in this group, 45 participants (58%) had at least one enhancing lesion.

**Figure 2:**
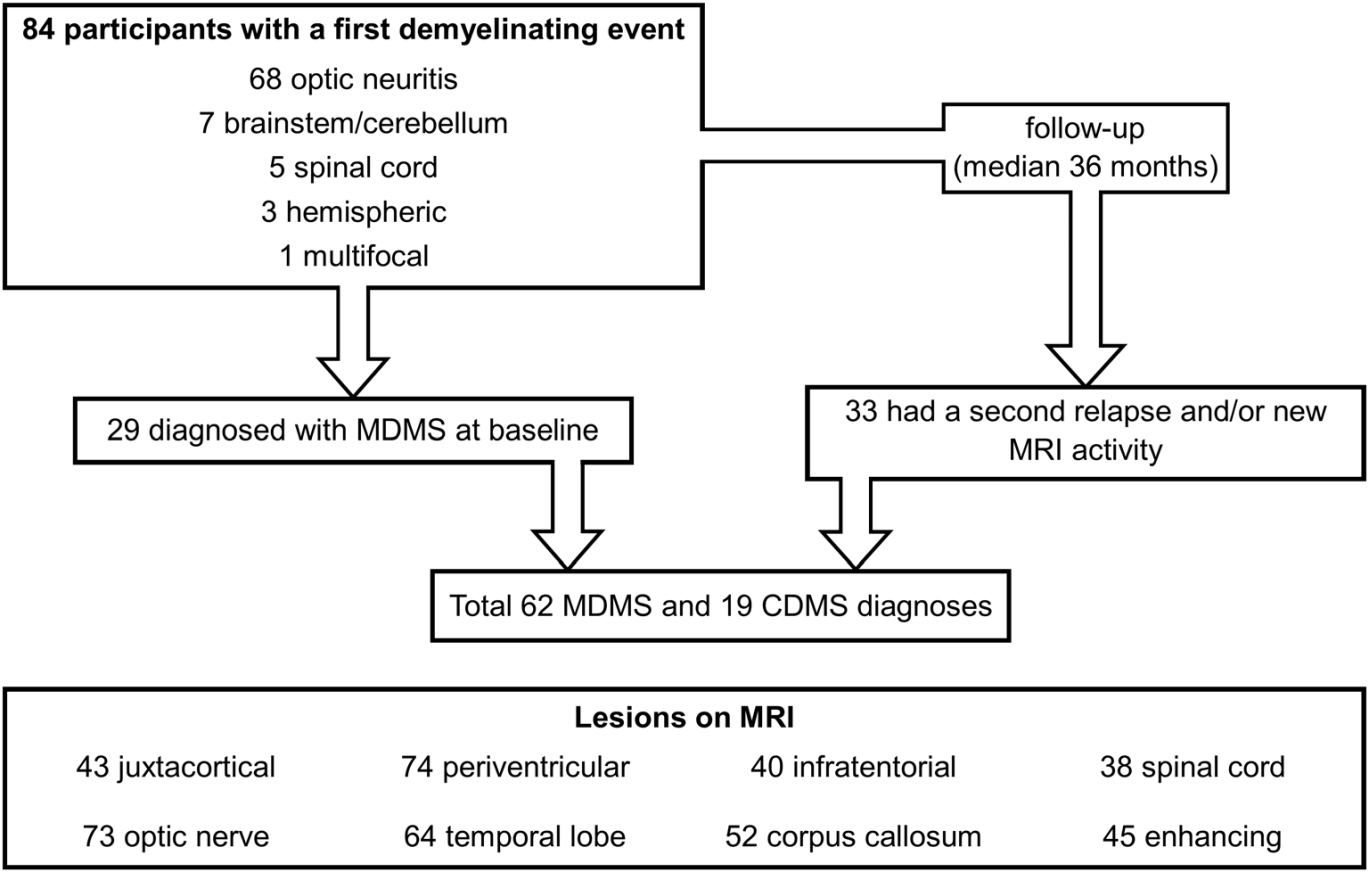
Flowchart showing the number of patients belonging to the different diagnostic categories; CDMS, clinically definite multiple sclerosis; MDMS, McDonald multiple sclerosis.

Twenty‐nine participants (35%) satisfied the 2017 McDonald criteria for MS at the time of baseline MRI, and 33 additional participants (39%) were diagnosed with MS over a median follow‐up of 36 months (interquartile range 48 months), with a final total of 62 participants (74%) with McDonald MS; 43 of the participants with McDonald MS were started on disease‐modifying treatment (69%). No participants with clinically isolated syndrome not satisfying criteria for McDonald MS diagnosis received disease‐modifying treatment. Median time to McDonald MS diagnosis (if not diagnosed at baseline) was 11.8 months (interquartile range: 9.3 months). Nineteen participants (23%) underwent CSF examination (at any point in follow‐up), with unmatched CSF oligoclonal bands identified in 12 of the tested participants (63%). Nineteen participants with McDonald MS (23% of total cohort, 31% of McDonald MS) experienced a second clinical relapse over follow‐up. A flowchart showing the number of patients belonging to the different categories is shown in Figure 2, while demographic, clinical, and MRI characteristics of the studied population are provided in Table 1.

**Table 1:** Demographic, clinical, and MRI characteristics of study participants.

Mean age, years (SD)	32.8 (7.1)
Sex, number of females	52
Median duration of follow‐up, months (IQR)	36 (48)
CSF examination (%)	19 (23)
MDMS at baseline (%)	29 (35)
MDMS through follow‐up (%)	33 (39)
CDMS through follow‐up (%)	19 (23)
MDMS on disease‐modifying treatment (%)	43 (69)
EDSS, median (range)	1 (0–3)
Achieva/Ingenia scanner	50/34
Contrast‐enhanced sequences (%)	78 (93)
Participants with juxtacortical lesions (%)	43 (51)
Participants with periventricular lesions (%)	74 (88)
Participants with infratentorial lesions (%)	40 (48)
Participants with spinal cord lesions (%)	38 (45)
Participants with optic nerve lesions (%)	73 (87)
Participants with corpus callosum lesions (%)	52 (76)
Participants with temporal lobe lesions (%)	64 (62)
Participants with enhancing lesions (%)	45 (54)

CDMS, clinically definite multiple sclerosis; EDSS, Extended Disability Status Scale; IQR, interquartile range; MDMS, McDonald multiple sclerosis; SD, standard deviation.

The existing DIS 2017 criteria were 87% sensitive (95% CI: 76–94), 73% specific (95% CI: 50–89%), and 83% accurate (95% CI: 74–91) when compared with the complete McDonald 2017 MS diagnostic criteria. The modified DIS criteria (requiring ≥2/5 regions) including either the optic nerve or the temporal lobe as a fifth anatomical location, as well as that with all the additional regions (optic nerve, temporal lobe and corpus callosum, requiring ≥3/7 regions), were significantly different compared with 2017 DIS criteria (*P* < 0.001, *P* = 0.004, and *P* = 0.004 respectively). The modified DIS criteria including the corpus callosum as a fifth anatomical location were statistically comparable (*P* = 0.4) to DIS 2017.

Among all modified DIS criteria, those including the optic nerve (≥2/5 regions) yielded the highest sensitivity (98%, 95% CI: 91–100) and the lowest specificity (33%, 95% CI: 13–59) with accuracy comparable to DIS 2017 criteria (84%, 95% CI: 74–91). The modified criteria with all the additional locations (≥3/7) showed the second highest sensitivity (95%, 95% CI: 87–99) and the highest accuracy (85%, 95% CI: 75–91). If more regions are required to meet the modified criteria including the optic nerve, specificity increased as sensitivity fell: when 3/5 regions are required, sensitivity was 82% (95% CI: 70–91), specificity 77% (95% CI: 55–92) and accuracy 81% (95% CI: 71–89); if 4/5 are required, sensitivity was 56% (95% CI: 43–69), specificity 95% (95% CI: 77–100) and accuracy 67% (95% CI: 56–77). Using the modified criteria with optic nerve as a fifth region (≥2/5 regions), six participants could have been diagnosed with MS at their first presentation with CIS, instead of the later diagnosis they obtained under the current criteria (amounting to 18% of the group diagnosed during follow‐up); with the seven‐region criteria (≥3/7 regions), four participants (12%) would have been diagnosed at presentation instead of during follow‐up. Diagnostic performances for 2017 DIS criteria and all modifications including one additional compartment are reported in Table 2. Performance of the modified criteria including the optic nerve where higher numbers of anatomical regions are required is reported in Table 3; performance of the seven‐region version is reported in Table S1.

**Table 2:** Diagnostic performance of the McDonald 2017 and modified DIS criteria adding one anatomical region for the development of relapsing–remitting multiple sclerosis according to the 2017 McDonald criteria (DIS + DIT).

	Sensitivity % (95% CI)	Specificity % (95% CI)	Accuracy % (95% CI)	PPV % (95% CI)	NPV % (95% CI)
DIS 2017 (≥2/4)	87 (76–94)	73 (50–89)	83 (74–91)	90 (82–95)	67 (50–80)
DIS+ON (≥2/5)	98 (91–100)	33 (13–59)	84 (74–91)	84 (79–88)	86 (44–98)
DIS+CC (≥2/5)	90 (80–96)	68 (45–86)	85 (75–91)	89 (81–94)	71 (53–85)
DIS+TL (≥2/5)	94 (84–98)	50 (28–72)	82 (72–90)	84 (78–89)	82 (49–89)

CC, corpus callosum; CI, confidence interval; DIS, dissemination in space; DIT, dissemination in time; NPV, negative predictive value; ON, optic nerve; PPV, positive predictive value; TL, temporal lobe.

**Table 3:** Diagnostic performance of the McDonald 2017 and modified DIS criteria with the optic nerve as an additional anatomical region for the development of relapsing–remitting multiple sclerosis according to the 2017 McDonald criteria (DIS+DIT).

	Sensitivity % (95% CI)	Specificity % (95% CI)	Accuracy % (95% CI)	PPV % (95% CI)	NPV % (95% CI)
DIS 2017 (≥2/4)	87 (76–94)	73 (50–89)	83 (74–91)	90 (82–95)	67 (50–80)
DIS+ON (≥2/5)	98 (91–100)	33 (13–59)	84 (74–91)	84 (79–88)	86 (44–98)
DIS+ON (≥3/5)	82 (70–91)	77 (55–92)	81 (71–89)	91 (82–96)	61 (46–73)
DIS+ON (≥4/5)	56 (43–69)	95 (77–100)	67 (56–77)	97 (84–100)	44 (37–51)

CI, confidence interval; DIS, dissemination in space; DIT, dissemination in time; NPV, negative predictive value; ON, optic nerve; PPV, positive predictive value.

**Figure 3:**
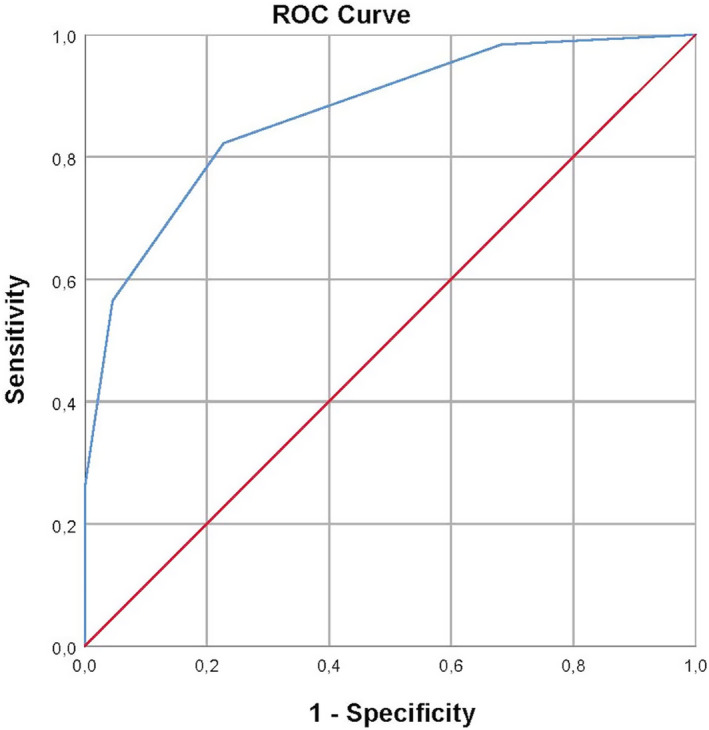
ROC curve showing the performance of demonstrating spatial dissemination (considering five possible compartments including the optic nerve) for the diagnosis of relapsing–remitting multiple sclerosis according to 2017 McDonald criteria. AUC is 0.87 (95% CI: 0.79–0.95).

With the ROC curve analysis for the modified criteria including the optic nerve (Fig. 3), the area under the curve (AUC) for diagnosis of MS was 0.87 (95% CI: 0.79–0.95). Additionally, in the ROC curve where all seven regions were included (Figure S1), AUC was 0.88 (95% CI: 0.80–0.96).

In combination with DIT criteria, the modified criteria including the optic nerve achieved the highest sensitivity (69%, 95% CI: 55–80) and lower specificity (63%, 95% CI: 38–84), with higher accuracy than the other combinations of DIS and DIT (68%, 95% CI: 56–78) (Table 4).

**Table 4:** Diagnostic performance of the McDonald 2017 and modified DIS criteria, plus DIT, for the development of relapsing–remitting multiple sclerosis according to the 2017 McDonald criteria (DIS+DIT) (*N* = 78).

	Sensitivity % (95% CI)	Specificity % (95% CI)	Accuracy % (95% CI)	PPV % (95% CI)	NPV % (95% CI)
DIS 2017 (≥2/4) and DIT	43 (30–57)	100 (82–100)	57 (43–68)	100 (86–100)	37 (32–42)
DIS+ON (≥2/5) and DIT	69 (55–80)	63 (38–84)	68 (56–78)	85 (76–91)	40 (28–53)
DIS+CC (≥2/5) and DIT	45 (32–58)	100 (82–100)	58 (47–70)	100 (87–100)	37 (32–43)
DIS+TL (≥2/5) and DIT	43 (30–57)	95 (74–100)	56 (44–67)	96 (78–99)	35 (30–41)

CC, corpus callosum; CI, confidence interval; DIS, dissemination in space; DIT, dissemination in time; NPV, negative predictive value; ON, optic nerve; PPV, positive predictive value; TL, temporal lobe.

## Discussion

Our main analysis demonstrates that the addition of new CNS regions to the existing DIS criteria, especially the optic nerve, increases sensitivity at diagnosing McDonald MS. While the inclusion of the optic nerve into the diagnostic criteria has been suggested previously,^9^ this study comprehensively assesses its contribution in a CIS population who all underwent dedicated optic nerve MRI. In a similar way to Vidal‐Jordana et al. for visual evoked potentials^12^ and Bsteh et al. for optical coherence tomography,^13^ this study demonstrates how optic nerve MRI can contribute to establishing optic nerve involvement, and therefore earlier diagnosis of MS. It also helps to address the inconsistency often seen in clinical practice, where people presenting with optic neuritis and lesions in one other CNS region on MRI are treated differently from those presenting with brainstem or spinal cord isolated syndromes and corresponding lesions. Additionally, for the first time, it examines the effect of different thresholds of affected anatomical regions on attainment of DIS when considering the optic nerve. With the growing number of highly effective disease‐modifying treatments for MS, and evidence demonstrating that early commencement of treatment improves long‐term outcomes,^32^, ^33^, ^34^ early diagnosis is becoming increasingly important.

While the optic nerve can be assessed with these different modalities, MRI carries certain advantages.^10^ Its accuracy is greater than the other modalities, and it is the only modality that allows visualization of the nerve itself, as opposed to markers of nerve integrity or function – this is particularly helpful to rule out compressive or infiltrative causes of optic neuropathies. Indeed, a well‐defined optic nerve lesion can help distinguish MS from mimics such as migraine and small‐vessel disease. It can be deployed anywhere in the time‐course of optic neuritis, rather than being restricted to use in either the acute or chronic phase (though it is particularly helpful in the acute phase, where swelling and gadolinium enhancement can be demonstrated).However, when evaluating the optic nerve, it is important to interpret any T2 hyperintensities in the context of the presenting syndrome, to reduce misdiagnosis of non‐MS optic neuropathies.

Various MRI sequences can be used to image the optic nerve. This study used two‐dimensional short tau inversion recovery (STIR), which offers high resolution and fat suppression; sensitivity is high for both acute (93%) and previous optic neuritis (94%).^35^ STIR sequences are acquired in 5–10 min, making it a clinically viable addition to standard brain and spine diagnostic imaging protocols, with high in‐plane resolution (0.5 × 0.5 mm). Three‐dimensional double‐inversion recovery (DIR) has alternatively been used for optic nerve imaging – it offers good contrast with fat and fluid suppression, though resolution is lower than STIR, acquisition time is longer, and it is vulnerable to air‐bone susceptibility artefacts. Sensitivity (95%) and specificity (94%) of DIR are high for identifying intrinsic optic nerve hyperintensities,^36^ superior to the performance of visual evoked potentials,^37^ and it has better sensitivity and specificity when compared against either 3D fluid‐attenuated inversion recovery (FLAIR)^38^ or 2D combined STIR‐FLAIR sequences.^36^ However, the tissue suppression provided by DIR may obscure soft‐tissue abnormalities compressing or infiltrating the optic nerve, which are more easily detectable by STIR. STIR therefore plays a helpful role in the differential diagnosis of optic neuritis‐like presentations (and consequently can contribute to the establishment of DIS in criteria including the optic nerve).

We acknowledge that the increase in sensitivity when adding the optic nerve as a fifth anatomical location comes at the expense of decreased specificity (and subsequently reduced accuracy). This is not unexpected when diagnostic criteria are expanded. However, it should also be noted that the analyzed cohort is highly controlled – clinical presentations were all suggestive of MS, and alternative diagnoses were excluded before inclusion in the cohort, as prescribed by the current 2017 guidelines. Additionally, the follow‐up duration is relatively short; previous studies reviewing the performance of modified criteria including the optic nerve had similar levels of specificity to this study at 36 and 60 months,^39^ whereas studies with longer follow‐up demonstrate better performance of McDonald criteria.^40^ These factors will conspire to reduce the observed specificity when DIT is a necessary criterion to diagnose MS. Indeed, as frequently stated, the McDonald criteria need judicious application to the clinical setting^2^: misdiagnosis is most likely when they are used inappropriately in presentations not suggestive of typical demyelination.^41^ We would therefore place greater emphasis on sensitivities rather than specificities in the interpretation of these modified criteria; this approach is likely to become increasingly relevant in future studies that investigate diagnostic criteria. This is particularly the case in clinical settings where early diagnosis and treatment are key – greater care might need to be taken in the recruitment for clinical trials. Where there is diagnostic uncertainty (even if criteria have been formally met), clinicians can increase confidence by looking for additional evidence of MS, such as DIT, the presence of CSF‐specific oligoclonal bands, or a higher degree of DIS.

It is also helpful to note how specificity increases as more anatomical locations are required (at the loss of sensitivity). The detection of demyelinating lesions in multiple regions on MRI after a single clinical episode is unlikely to suggest a single moment of inflammatory activity. Rather, it may instead be reflective of sub‐clinical demyelination occurring at timepoints prior to the clinical event – what might have been a radiologically isolated syndrome had imaging been performed prior to the first clinical presentation. A higher and more disseminated number of lesions (as a surrogate marker for DIT) could therefore more strongly suggest MS than a modest lesion load. Indeed, specificity is further improved when observed DIT (heterogenous enhancement of lesions on initial MRI, new T2‐hyperintense lesions, new relapse, presence of oligoclonal bands) is added to the modified criteria. A post hoc analysis distinguishing optic neuritis from other presentations showed that the modified DIS criteria have no impact on nonoptic neuritis presentations (Table S3), also previously observed by Brownlee et al.^11^ However, the nonoptic neuritis group was small (16/84 participants, 19%) and therefore relatively under‐represented.

The analysis compares modified diagnostic criteria with the 2017 revisions of the McDonald criteria,^2^ rather than against a diagnosis of “clinically definite multiple sclerosis.”^15^ The latter label, which relies on the occurrence of at least two clinical relapses, has largely been discarded from modern diagnostic criteria. Many people with a diagnosis of MS by McDonald criteria may not experience a second relapse for many years (if at all), as highly effective treatment is started early with the express aim of preventing further relapses. The people in our cohort were treated under this approach – of 62 participants diagnosed with McDonald MS, 43 (69%) were started on disease‐modifying treatment – and hence very few achieved the “clinically definite” status. It is possible that more might do so with a longer duration of follow‐up, of course, but the McDonald criteria^2^ were more suited for use as the current gold‐standard for diagnosing MS.

The relatively short median follow‐up is a limitation of this study: a longer duration might have revealed a higher rate of relapsing–remitting MS diagnosis, and an increased ability to compare modified criteria to standard McDonald diagnosis. Additionally, CSF examination was requested when there was a clinical indication in cases who did not fulfil DIT but had DIS, which would be the routine diagnostic pathway (rather than as a study requirement). Six participants meeting DIS criteria at baseline had not demonstrated DIT by the end of the follow‐up period; of these, one had a negative CSF examination, three declined the procedure, and two withdrew from the study before CSF examination could be performed. Furthermore, five participants with DIS at baseline demonstrated DIT through follow‐up, precluding the need for CSF examination.

Another feature of this cohort is the over‐representation of optic neuritis presentations: 81% of participants presented with optic neuritis, compared with reported frequencies of 18–32%.^42^, ^43^ Other analyses with similar proportions of optic neuritis presentations have been published elsewhere,^11^, ^40^ including one assessing validity of a previous iteration of the McDonald criteria.^44^ This bias might also have contributed to the loss of specificity with the modified criteria, as people presenting with an optic neuritis clinically isolated syndrome may be less likely to convert to MS (though this lower risk is mitigated once cranial MRI at baseline is accounted for)^45^; a more heterogenous cohort might have had more rapid diagnosis of McDonald MS. However, this does provide a substantial cohort of optic nerve presentations in which to test modified criteria with optic nerve lesion involvement. It is also important to note that, while optic neuritis clinically isolated syndrome may be less likely to convert to MS, people with MS who initially presented with an optic neuritis demonstrate better long‐term outcomes when disease‐modifying treatments are started earlier.^46^

Overall, our results indicate that adding the optic nerve to the criteria for determining DIS as a fifth CNS compartment would increase sensitivity while reducing specificity (and preserving accuracy) when two out of five regions are required. Increasing the number of required regions (e.g., 3/5 or 4/5 where the optic nerve is the fifth region) increases specificity and might provide an alternate pathway to MS diagnosis. Alternatively, modifications adding all three proposed compartments (optic nerve, corpus callosum, and temporal lobe) to the current four, and requiring involvement of three of seven compartments, would still increase sensitivity while better preserving specificity (and also accuracy). However, this approach would be more laborious, with potential for confusing overlap between the new regions and spatial misclassification (e.g., a lesion in the corpus callosum abutting a ventricle being misclassified as periventricular instead of callosal). In either case, optic nerve involvement has an important role to play in the diagnosis of MS.

## Author Contributions

AT, CGWK, FB, OC, and WB contributed to conception and design of the study. Acquisition and analysis of data were performed by AB, BK, FB, FP, GP, ID, LO, MF, MY, and SC. Manuscript and figure preparation were undertaken by AT, FB, GP, MF, and OC.

## Conflicts of Interest

MF has received speaker honoraria from Merck. SC has received speaker honoraria from Merck. OC is a member of independent DSMB for Novartis, gave a teaching talk on McDonald criteria in a Merck local symposium, and contributed to an Advisory Board for Biogen. WB has received speaker honoraria for educational activities and/or acted as a consultant for Biogen, Janssen, Merck, Novartis, Roche, Sanofi‐Genzyme, and Viatris. FB is a consultant to Bayer, Biogen, Roche, Merck‐Serono, Novartis, and Janssen. AT has received speaker honoraria from Biomedia and Merck and meeting expenses from Biogen Idec and Merck, and was the UK PI for two clinical trials sponsored by MEDDAY pharmaceutical company (MD1003 in optic neuropathy [MS‐ON – NCT02220244] and progressive MS [MS‐SPI2 – NCT02220244]). The pharmaceutical companies listed manufacture medicines used to treat multiple sclerosis, which may be prescribed more frequently if more diagnoses of multiple sclerosis are made. The other authors have no relevant interests to declare.

## Supporting information


**Data S1.** Supplementary materials.
**Table S1.** Diagnostic performance of the McDonald 2017 and modified DIS criteria including all seven compartments for the development of relapsing‐remitting multiple sclerosis according to the 2017 McDonald criteria (DIS+DIT).
**Table S2.** Diagnostic performance of the McDonald 2017 and modified DIS criteria for the development of relapsing‐remitting multiple sclerosis according to the 2017 McDonald criteria (DIS+DIT) in patients presenting with an optic neuritis (*N* = 68).
**Table S3.** Diagnostic performance of the McDonald 2017 and modified DIS criteria for the development of relapsing‐remitting multiple sclerosis according to the 2017 McDonald criteria (DIS+DIT) in patients with presentations other than optic neuritis (*N* = 16).
**Figure S1.** ROC curve showing the performance of demonstrating spatial dissemination (considering all seven possible compartments) for the diagnosis of relapsing‐remitting multiple sclerosis according to the 2017 McDonald criteria. AUC is 0.88 (95% CI: 0.80–0.96).

## Data Availability

The data that support the findings of this study are available on request from the corresponding author. The data are not publicly available due to privacy or ethical restrictions.
