# Supplementary material for: Improving criteria for dissemination in space in multiple sclerosis by including additional regions
Source: Ann Clin Transl Neurol. 2024 Jul 30;11(10):2572–82. doi: 10.1002/acn3.52170 (PMC11514922; doi:10.1002/acn3.52170)
Supplement: Supplementary file 1 — Data S1. Supplementary materials. Table S1. Diagnostic performance of the McDonald 2017 and modified DIS criteria including all seven compartments for the development of relapsing‐remitting multiple sclerosis according to the 2017 McDonald criteria (DIS+DIT). Table S2. Diagnostic performance of the McDonald 2017 and modified DIS criteria for the development of relapsing‐remitting multiple sclerosis according to the 2017 McDonald criteria (DIS+DIT) in patients presenting with an optic neuritis (N = 68). Table S3. Diagnostic performance of the McDonald 2017 and modified DIS criteria for the development of relapsing‐remitting multiple sclerosis according to the 2017 McDonald criteria (DIS+DIT) in patients with presentations other than optic neuritis (N = 16). Figure S1. ROC curve showing the performance of demonstrating spatial dissemination (considering all seven possible compartments) for the diagnosis of relapsing‐remitting multiple sclerosis according to the 2017 McDonald criteria. AUC is 0.88 (95% CI: 0.80–0.96). [file ACN3-11-2572-s001.docx]

**Supplementary materials**

## **Methods**

The brain MRI protocol included 3D gradient echo T1-weighted (acquisition time (AT) = 6:35 min, voxel-size = 1x1x1 mm, flip angle (FA) = 8°, sagittal acquisition; Achieva: repetition time (TR) = 6.8 ms, echo time (TE) = 3.0 ms; Ingenia: TR = 7.0 ms, TE = 3.2 ms), 3D fluid-attenuated inversion recovery (FLAIR) (AT = 6:35 min, sagittal acquisition; Achieva: voxel-size = 1.2x1.2x1.2 mm, TR = 8000 ms, TE = 394 ms, inversion time (TI) = 2400 ms; Ingenia: voxel-size: 1x1x1 mm, TR = 5000 ms, TE = 350 ms, TI = 1650 ms), 2D-axial conventional spin echo proton density (PD)/T2-weighted (AT = 3:41 min, voxel-size = 1x1x3 mm, TR = 3500 ms, TE = 19/85 ms), and 2D-axial pre- and post-contrast conventional spin echo T1-weighted (AT = 5:43 min, voxel-size = 1x1x3 mm, TR = 625 ms, TE = 10 ms) sequences.

The optic nerve MRI protocol comprised 2D-coronal short tau inversion recovery (STIR, AT = 6:21 min, voxel-size = 0.8x0.8x2.0 mm, TR = 4646 ms, TE = 75 ms, TI = 200 ms), and post-contrast 2D fat-saturated conventional spin echo axial (AT = 5:33 min, voxel-size = 0.7x0.9x2.0 mm, TR = 400 ms, TE = 12 ms) and gradient echo coronal (AT = 6:47 min, voxel-size = 0.8x0.8x3.0 mm, FA = 25°, TR = 45 ms, TE = 2.6 ms) T1-weighted sequences.

The spinal cord MRI protocol acquired sagittal images over a 480 mm field-of-view to cover the whole cord with 1x1x3 mm voxel size. It included 2D fast spin-echo sagittal PD-weighted (AT = 5:43 min; Achieva: TR = 3500 ms, TE = 21 ms; Ingenia: TR = 3500 ms, TE = 31 ms), T2-weighted (AT = 5:20 min; Achieva:, TR = 3900 ms, TE = 76 ms; Ingenia: TR = 3900 ms, TE = 70 ms), and pre- and post-contrast T1-weighted spin-echo (AT = 4:53 min, TR = 600 ms, TE = 8 ms) scans. No axial cord images were acquired.

## **Tables**

**Table S1:** Diagnostic performance of the McDonald 2017 and modified DIS criteria including all seven compartments for the development of relapsing-remitting multiple sclerosis according to the 2017 McDonald criteria (DIS+DIT)

|  | **Sensitivity**  % (95% CI) | **Specificity**  % (95% CI) | **Accuracy**  % (95% CI) | **PPV**  % (95% CI) | **NPV**  % (95% CI) |
| --- | --- | --- | --- | --- | --- |
| DIS 2017 (≥2/4) | 87 (76-94) | 73 (50-89) | 83 (74-91) | 90 (82-95) | 67 (50-80) |
| DIS+ON+CC+TL (≥3/7) | 95 (87-99) | 55 (32-76) | 85 (75-91) | 86 (79-90) | 80 (55-93) |
| DIS 2017 (≥2/4) and DIT | 43 (30-57) | 100 (82-100) | 57 (43-68) | 100 (86-100) | 37 (32-42) |
| DIS+ON+CC+TL (≥3/7) and DIT | 66 (52-78) | 74 (49-91) | 68 (56-78) | 88 (78-94) | 41 (31-52) |

**Table S2:** Diagnostic performance of the McDonald 2017 and modified DIS criteria for the development of relapsing-remitting multiple sclerosis according to the 2017 McDonald criteria (DIS+DIT) in patients presenting with an optic neuritis (N = 68)

|  | **Sensitivity**  % (95% CI) | **Specificity**  % (95% CI) | **Accuracy**  % (95% CI) | **PPV**  % (95% CI) | **NPV**  % (95% CI) |
| --- | --- | --- | --- | --- | --- |
| DIS 2017 (≥2/4) | 84 (70-93) | 74 (49-91) | 81 (70-89) | 89 (79-97) | 64 (47-78) |
| DIS+ON (≥2/5) | 98 (89-100) | 21 (6-46) | 76 (65-86) | 76 (72-80) | 80 (32-97) |
| DIS+CC (≥2/5) | 88 (75-95) | 68 (43-87) | 82 (71-91) | 88 (79-93) | 68 (49-83) |
| DIS+TL (≥2/5) | 92 (80-98) | 53 (29-76) | 81 (70-89) | 83 (76-89) | 71 (47-88) |
| DIS+ON+CC+TL (≥3/7) | 94 (83-99) | 53 (29-76) | 82 (71-91) | 84 (76-89) | 77 (51-92) |

**Table S3:** Diagnostic performance of the McDonald 2017 and modified DIS criteria for the development of relapsing-remitting multiple sclerosis according to the 2017 McDonald criteria (DIS+DIT) in patients with presentations other than optic neuritis (N = 16)

|  | **Sensitivity**  % (95% CI) | **Specificity**  % (95% CI) | **Accuracy**  % (95% CI) | **PPV**  % (95% CI) | **NPV**  % (95% CI) |
| --- | --- | --- | --- | --- | --- |
| DIS 2017 (≥2/4) | 100 (75-100) | 67 (9-99) | 94 (70-100) | 93 (72-98) | 100 (16-100) |
| DIS+ON (≥2/5) | 100 (75-100) | 67 (9-99) | 94 (70-100) | 93 (72-98) | 100 (16-100) |
| DIS+CC (≥2/5) | 100 (75-100) | 67 (9-99) | 94 (70-100) | 93 (72-98) | 100 (16-100) |
| DIS+TL (≥2/5) | 100 (75-100) | 33 (1-91) | 88 (62-98) | 87 (74-94) | 100 (3-100) |
| DIS+ON+CC+TL (≥3/7) | 100 (75-100) | 67 (9-99) | 94 (70-100) | 93 (72-98) | 100 (16-100) |

CC = corpus callosum; CI = confidence interval; DIS = dissemination in space; DIT = dissemination in time; NPV = negative predictive value; ON = optic nerve; PPV = positive predictive value; TL = temporal lobe

## **Figure**


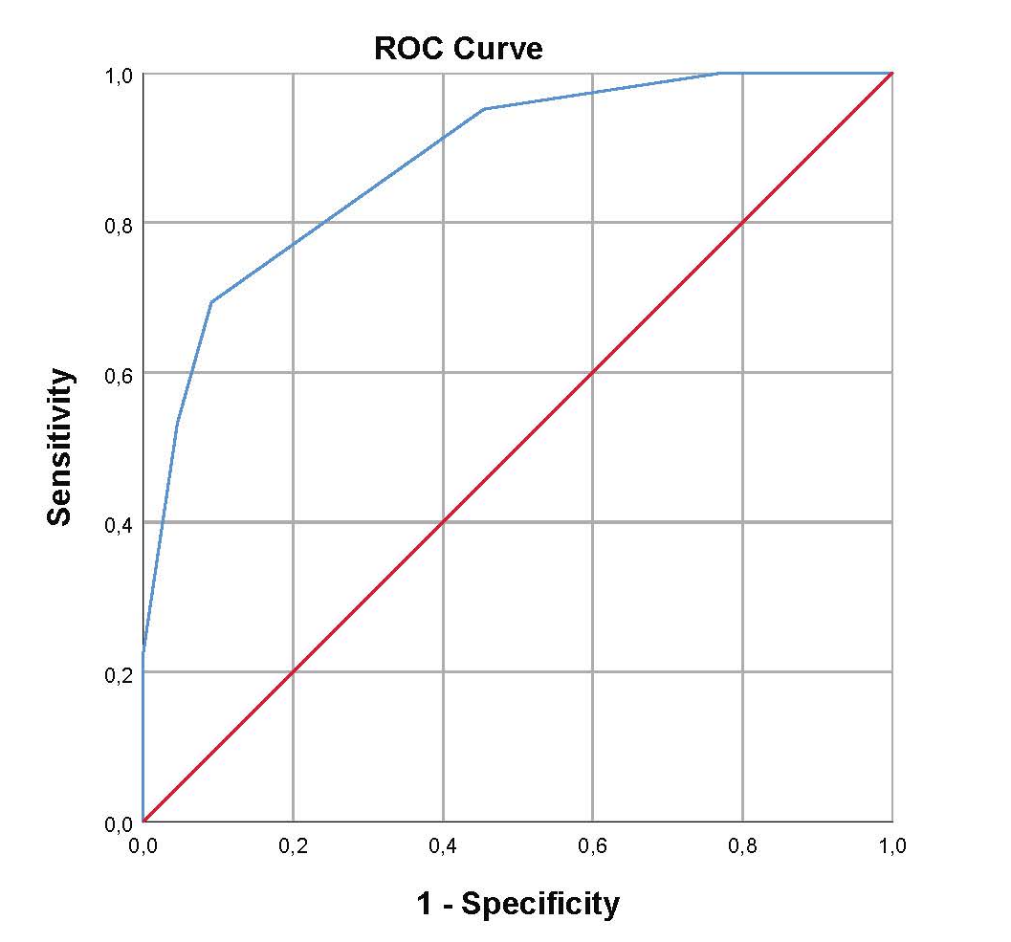


**Figure S1:** ROC curve showing the performance of demonstrating spatial dissemination (considering all seven possible compartments) for the diagnosis of relapsing-remitting multiple sclerosis according to the 2017 McDonald criteria. AUC is 0.88 (95% CI 0.80-0.96)
